# A retrospective chart review to assess the impact of alpha-guided transcranial magnetic stimulation on symptoms of PTSD and depression in active-duty special operations service members

**DOI:** 10.3389/fpsyt.2024.1354763

**Published:** 2024-06-21

**Authors:** Marybeth Bailar-Heath, Riley Burke, Delisha Thomas, Chad D. Morrow

**Affiliations:** Human Performance Optimization Department, Brain Health Clinic, Air Force Special Operations Command (AFSOC) Geographically Separated Unit (GSU), Fayetteville, NC, United States

**Keywords:** military medicine, PTSD, TMS, individualized alpha peak TMS, TBI, special operations forces, MeRT

## Abstract

**Introduction:**

Special Operations Forces service members (SOF) are regularly exposed to traumatic and concussive events, increasing the prevalence of symptoms of post-traumatic stress disorder (PTSD) and depression, shortening potential years of service.

**Methods:**

This retrospective chart review presents preliminary data on a Human Performance Optimization (HPO) program that provided an average of 30 sessions of individualized alpha frequency repetitive transcranial magnetic stimulation (α-rTMS) to active-duty SOF as to reduce symptoms of PTSD and depression following traumatic brain injury. Scores from the PTSD Checklist for DSM-5, PROMIS Depression short form and Perceived Deficits Questionnaire (PDQ) were reviewed.

**Results:**

Significant reductions were noted after the HPO program in all clinical scales with an average 37% decrease in PCL-5 (*p*<.01), 11.3% reduction in PROMIS depression T-scores (*p*<.01), and 45.5% reduction in PDQ scales by session 30 (*p*<.01), with side effects matching those commonly reported in rTMS. Importantly, the average PCL-5 score decreased from 42.9 to 27 by end of the treatment program, which is below the clinical threshold of 33 for presence of PTSD. For those with depression symptoms scores greater than cut off clinical thresholds at baseline, 46% resolved following treatment.

**Conclusion:**

This data provides preliminary support for safe application of α-rTMS for symptom reduction in active-duty special operations military personnel.

## 1 Introduction

Special Operations Forces service members (SOF) operate in dynamic environments and frequently experience psychological traumas, concussive forces, and blast waves (1–5). According to the US Department of Veteran’s Affairs (VA), of veterans who served in Iraq and/or Afghanistan receiving VA services in 2021, up to 24% met diagnostic criteria for post-traumatic stress disorder (PSTD) (6). In comparison to conventional forces, SOF generally engage in a healthier lifestyle (7) yet they have higher levels of traumatic brain injury (TBI) due to more rigorous training, intensity and number of deployments (8). As SOF have an increased risk of TBI when compared to conventional forces (9), they also have higher prevalence of multimorbidity, as military personnel with TBI are 4.18 times more likely to have PTSD than personnel without TBI (10). Depression is also prevalent in the armed services, with a recent meta-analysis revealing a 23% prevalence of depression in active duty military (11).

PTSD and depression are characterized by alterations in cognition and mood, and both disorders present with impairments in executive function, attentional control and working memory (12, 13). These cognitive processes are engaged through interconnected large-scale cortical networks, specifically the Default Mode Network (DMN), the predominant resting-state network, which has been shown to be impaired in patients with PTSD (14). PTSD has been characterized by a general lack of prefrontal connectivity, with decreased medial prefrontal cortex (mPFC) connectivity, and frontal DMN connectivity inversely correlated with increasing symptoms of PTSD (14, 15). Furthermore, in instances of comorbid TBI and PTSD, although not directly measured in this present study, network connectivity has also been reported to be inversely correlated with PTSD severity, with significantly lower connectivity noted in the mPFC in co-occurring conditions as compared to those with TBI alone (16).

The human alpha rhythm, which is the predominant resting-state cortical rhythm, with oscillations occurring between 8-13Hz (17), has been reported to be closely linked to DMN activity (18–20). Alpha activity is clustered around a single frequency, the individual alpha frequency (IAF), which peaks in frequency in early adulthood, and slows with age (21), with overlapping sites of generation of alpha and DMN activity in frontal and parietal regions (18). IAF is associated with key cognitive and emotional functions, including working memory, visual perception, reaction speed, time perception, emotional memory consolidation, and likely representative of DMN health via synchronization of spontaneous network activity and suppression of cortical propagation (22, 23). Disruptions in alpha synchronization are reported in affective and anxiety disorders (24), as well as in PTSD (25), with deficits in alpha networks theorized to correspond with symptoms of hypervigilance, sensory disinhibition, and alterations in working memory present in PTSD. Neuromodulation, in the form of repetitive transcranial magnetic stimulation (rTMS) has been shown to decrease hyperconnected DMN networks reported in depression (26), and when applied as transcranial alternating current stimulation targeting alpha oscillations in healthy controls has been shown to upregulate local alpha EEG power and increase DMN connectivity (27). Additionally, rTMS delivered at IAF (α-rTMS) has been preliminarily reported to improve clinical response in PTSD (28). Symptom response to neuromodulation has been correlated with stimulation frequency proximity to IAF in depression (29–31), making it a promising interventional approach for use in the PTSD population.

In the past decade, personalized noninvasive neuromodulation targeting disrupted networks has been explored for the treatment of depression and PTSD in military populations (28, 32, 33). The most common side effects of rTMS include headache, pain at the site of stimulation, and nausea resolving within several therapy sessions (34). For the Human Performance Optimization (HPO) program studied here, physicians used α-rTMS for active-duty SOF personnel with a history of TBI who suffered from symptoms of depression and PTSD and consequently were at risk for development of long-term psychiatric illness. α-rTMS was used in the HPO program for its specific targeting of disrupted neural circuits and function implicated in PTSD and TBI in comparison to 10Hz rTMS or intermittent theta burst stimulation (iTBS). In this retrospective chart review, active-duty SOF who participated in this HPO program that deployed α-rTMS were identified and reviewed, and preliminary safety and clinical response data is reported.

## 2 Materials and methods

### 2.1 Clinical population

Charts from active-duty SOF who had received α-rTMS as part of an HPO program were identified for review. The HPO program was run in a US-based outpatient military Brain Health Clinic and focused specifically on improving symptoms of persistent post-concussive syndrome. Potential participants learned of the Program through email and via conversation as a performance and optimization program. Participation in the HPO program was voluntary, and senior leadership was not involved in program recruitment. HPO program inclusion for the active-duty SOF included a self-report of history of head injury obtained by a neuropsychologist from a full history taken at screening, and capability to commit to 5 days of α-rTMS sessions per week for 6 weeks (30 sessions in total). Program participants were not formally assessed for clinical diagnoses. In this chart review clinical symptoms scales, which contain cutoffs suggestive of clinical diagnosis, were used to monitor participant’s self-reported symptom severity at baseline, midpoint and end of the HPO program. Clinical scales used in the HPO program include the PCL-5 to assess PTSD symptom severity (35), the PROMIS Depression short form, used to assess depression symptom severity (36), and the Perceived Deficits Questionnaire (PDQ), which measures self-inferential assessment data (37).

Inclusion required approval from unit command leadership, command psychologist and the physician medical director. Program exclusion criteria included pacemakers, defibrillators, any metal in the head, history of seizures, as well as current benzodiazepine or alcohol use. Participants were asked daily to report on sleep quality, and any possible side effects, including presence of headaches, fatigue, and pain, were noted by staff providing the α-rTMS. HPO program participants provided informed written consent, which included notification that α-rTMS was off-label.

Charts of SOF from the Air Force Special Operations Command Geographically Separated Unit (AFSOC GSU) at an outpatient medical facility who had undergone the HPO program and received α-rTMS between January 2020 and August 2021, were reviewed. Patient charts were accessed, with information kept in paper and electronic charts aggregated. Prior to analysis and during data aggregation, all identifying patient information was removed and deidentified. Charts were examined for data pertaining to intake and screening notes, clinical notes, history of injury, clinical scales, demographics, length of service, as well as daily therapy notes taken throughout therapy. It was noted that medications and concomitant therapies were not available for review. Additional charts search keywords included: sleep, pain, anxiety and depression, headache. Side effects from α-rTMS were also searched for within clinical and daily therapy notes.

### 2.2 α-rTMS procedure

α-rTMS (known commercially as Magnetic E-Resonance Therapy (MeRT)) was delivered five days per week for 6 weeks with a MagVenture MagPro R30 stimulator and b65 coil, with stimulation frequency at patient-measured IAF, and target locations of midline frontopolar (Fpz) and midline parietal (Pz) regions with rTMS coil orientation perpendicular to the midline. EEGs were recorded with a Deymed Truscan EEG system and fitted 21-lead FlexiCAP with standardized 10-20 positioning. A 10-minute eyes closed EEG recording was obtained on patients biweekly. Each participant’s EEG data was processed by the MeRT algorithm, which calculates and identifies α-rTMS frequency parameters as the dominant EEG alpha peak frequency in the 8-13Hz range in a posterior-occipital region of interest (ROI) consisting of P3, P4, Pz, O1, and O2 electrodes. Patient IAF was determined following a fast-fourier transform (FFT) of the posterior ROI on data following initial EEG automated artifacting. Resting motor threshold (rMT) was measured prior to receiving α-rTMS as the minimum stimulation intensity delivered to bilateral motor cortical regions that elicits a contralateral thumb twitch as visually evaluated by a trained technician. Starting stimulation site was the bilateral prefrontal cortex, over the Fpz EEG site. Stimulation was delivered at IAF in 5-second trains, with 45 second intertrain intervals, for 36 trains, lasting approximately 30 minutes for patients receiving a single site of stimulation. Patients with poor posterior alpha peak synchronization after the first EEG had the addition of stimulation to the bilateral central parietal region, with the coil centered at the Pz EEG site. Poor alpha synchronization was identified by the presence of a low-amplitude, broad based posterior alpha peak reflective of high alpha frequency variability around IAF. Subjects receiving stimulation at two cortical sites received the same 5 seconds of stimulation, with 28-second intervals and 32 trains per site. IAF was recalculated biweekly, with intensity at no more than 80% of patient rMT to minimize side effects. Clinical scales were administered by clinicians prior to the first α-rTMS session, at three weeks, and at the final session. Daily therapy notes were also retrieved.

### 2.3 Statistical analysis

Session information, α-rTMS parameters, clinical scales and notes were gathered from patient charts at the commencement of the review. Analysis of variance was first used to test for a main effect of time across the three repeated measures for each scale. Paired samples T-tests were then performed on data taken prior to their first α-rTMS session versus their midpoint, or final session data, with *p*<0.05 considered statistically significant. Hedge’s *g* (38) was calculated to determine the effect size for a change from baseline on a given psychometric scale. Remission rates were calculated on subjects with a baseline score above the threshold for the scale. The clinical threshold for the PCL-5 was 33, and 55 for the PROMIS T-score. Clinically relevant scale reductions are 10 points for PCL (39) and 3 points for the PROMIS T-score (40). Data were plotted to show the mean with error bars at +/- one standard error of the mean.

## 3 Results

Eighteen participants’ charts were found, and seventeen males (mean age of 39.2, range 33-55, SD=5.3) and one 33-year-old female were reviewed. SOF personnel had served an average of 17.3 years (range 11-26, SD=4.7). No SOF had any current or previous diagnoses of PTSD or depression.

SOF received an average of 30.9 α-rTMS sessions (20-53, SD=1.4), with average stimulation frequency assigned by the MeRT algorithm of 10.8Hz (range 9.6-12.3, SD=0.65). Average mid and endpoint frequency settings were 10.8Hz (9.6-12.3, SD=0.64) and 10.7Hz (9.5-12, SD=0.59). There were no significant differences in treatment frequency calculation by time (*F*_2,34_ = 1.1, *p*=.35), and *post-hoc* pairwise comparisons were nonsignificant (*p*>.05). All SOF started with one stimulation location at the Fpz site, with 5 switching to stimulation at Pz and Fpz sites following the midpoint EEG. There was no significant difference in frequency of stimulation between dual site subjects and single site at baseline (*p*=.14) or end of program (*p*=.56), however stimulation frequency for the dual site group was higher at baseline (11.2Hz versus 10.7) and end of program (10.8 versus 10.6).

With respect to commonly reported side effects of the α-rTMS program, at the end of 20 treatment sessions (the minimum number of treatment sessions with complete data for all participants) there was one participant who reported headache (5.5%), and one participant who reported nausea/vertigo (5.5%). No other side effects were reported at the conclusion of 20 treatment sessions. It was noted that some side effects were experienced transiently throughout the sessions, with significantly fewer in the last ten treatment sessions in comparison to the first ten sessions. For example, 33% (6 of 18 participants) reported headaches at least two times throughout the first ten treatment sessions, of which all but one resolved during the last ten sessions; and 39% (7 of 18 participants) reported insomnia/fatigue during the first ten treatment sessions, of which all but three resolved during their last ten sessions. No SAE (serious adverse event) was reported in charts.

It was noted in the charts reviewed, that two participants reported a single instance of suicidal ideation (1 participant at only session 5, and 1 participant at only session 3). Both were cleared to continue treatment as per established clinic protocol as these instances were not considered related to the treatment, and neither reported any other than the single occurrences.

**Figure 1:**
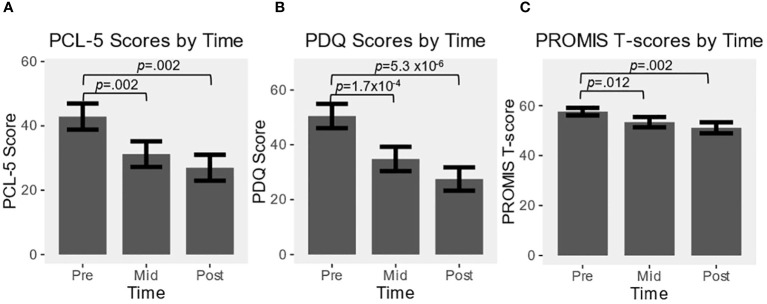
Effect over time of alpha guided neuromodulation (alpha-rTMS) in program participants for: **(A)** PCL-5; **(B)** PDQ; **(C)** PROMIS. Values are represented as mean ± SEM. All pre vs. mid, and pre vs. post comparisons were statistically significant at *p*<0.05.

All baseline and mid-point scales were completed, with one subject not completing final scales. A significant main effect of time was detected for all three scales (PCL-5 *F*_2,33_ = 12.1, *p*<.001; PROMIS *F*_2,33_ = 8.8, *p*<.001; PDQ *F*_2,33_ = 28.9, *p*<.001), and significant reductions from baseline were found at mid- and end-points for all scales (**Figure 1**).

The average PCL-5 baseline score was 42.9 (15-72, SD=16.8), midpoint of 31.2 (7-58, SD=16.3), and final score of 27 (4-52, SD=16.1). PCL-5 reduction was significant at mid (*t_17_ =* 3.70, *p*=.002, *g*=.83) and end-points (*t_16_ =* 3.68, *p*=.002, *g*=.89). Twelve SOF personnel (67%) had data at all time points and reported a PCL-5 score of greater than the PTSD cutoff of 33 at baseline, with an average score of 51 (35-72, SD=12.6). The score dropped significantly over the course of the program, to an average of 35.8 (11-58, SD=13.1) by midpoint (*t_11_ =* 4.21, *p*=.001, *g*=1.1) and 32.8 (8-52, SD=13.8) by the end (*t_11_ =* 3.47, *p*=.005, *g*=.93), with 5 of 12 patients (42%) scoring under 33 at completion, and 8 total participants decreased by a clinically relevant amount of 10 or greater.

The average baseline PROMIS T-score was 57.6 (46.2-76.2, SD=6.4), reduced to 53.4 at midpoint (37.1-65.4, SD=8.8), and final score of 51.1 (37.1-63.5, SD=9.2). PROMIS score reduction was significant at both mid (*t_17_ =* 2.82, *p*=.012, *g*=.67) and end-points (*t_16_ =* 3.61, *p*=.002, *g*=.88). Thirteen SOF personnel had data at all time points and reported a PROMIS score greater than 55 at baseline (72%), with an average score of 59.9 (55.3-76.2, SD=5.8). The score dropped significantly over the course of the program, to an average of 56.1 (43.3-65.4, SD=6.6) by midpoint (*t_12_ =* 2.33, *p*=.04, *g*=.61) and 55 (37.1-63.5, SD=6.5) by the end (*t_12_ =* 2.30, *p*=.04, *g*=.60), with 6 of 13 patients (46%) scoring under 55 at completion, and 7 of 13 (54%) total participants decreased by a clinically relevant amount.

The average PDQ baseline score was 50.5 (12-80, SD=18.3), midpoint score of 34.8 (5-69, SD=16.3), and final average score of 27.5 (4-66, SD=16.1). PDQ score reduction was significant at both mid (*t_17_ =* 4.79, *p*=1.7x10^-4^, *g*=1.08) and end-points (*t_16_ =* 6.68, *p*=5.3x10^-6^, *g*=1.54).

## 4 Discussion

Special operations forces service members are at greater risk for TBI as compared to conventional forces (8), which represents an increased risk for TBI comorbid conditions, often including PTSD and depression. TBI has been demonstrated to significantly impair stability of the DMN (41), which has been shown to be affected in patients with depression and PTSD. In this retrospective review, we examined charts from healthy active-duty operators with a history of TBI who participated in a Human Performance Optimization (HPO) program that utilized guided neuromodulation in the form of α-rTMS. The personalized noninvasive neuromodulation used in this program targets disrupted networks, and in the present chart review, the HPO program resulted in decreased severity of PTSD and depression symptoms, with 42% and 46% of participants no longer meeting severity thresholds for PTSD or depression, respectively. Recorded side effects in this population were equivalent to or slightly elevated compared to side effects reported in application of rTMS in depression. Headache is generally the most common side-effect of rTMS, with one study of 412 patients receiving 10Hz rTMS or intermittent theta burst TMS reporting 64% and 65% incidence of headache, respectively (42). At the end of 20 treatment sessions there was one participant who reported headache (5.5%), and one participant who reported nausea/vertigo (5.5%). No other side effects were reported at the conclusion of 20 treatment sessions. While there were transient reports of headaches (33%), all resolved with the exception of one participant (5.5%). Additionally, while there were transient reports of fatigue and insomnia, which are generally elevated in active-duty military populations, as it has been reported that as few as 27-30% of active-duty soldiers obtain the recommended minimum of 7 hours of sleep per night for adults (43, 44), these symptoms resolved by the end of the treatment sessions. Other side effects matched those normally reported in TMS application, with resolution of most side effects by the second half of the program.

In current practice, rTMS is most often provided as a therapeutic intervention to patients who are suffering from diagnosed clinical conditions, and is generally provided in military populations after they complete their service. The military has implemented programs that attempt to combat the development and severity of PTSD and depression, including the deployment of cognitive behavioral therapy, collaborative care interventions and virtual-reality-based interventions, however none directly target and interact with potentially disrupted neurocircuitry (45). The reduction in PTSD and depression symptoms illustrated in the current chart review may represent a relative reduction in risk for further development of these disorders, however long-term follow-up with a comparison group is necessary for such conclusions.

As this was a retrospective chart review and not a prospective trial, conclusions are limited by low sample size, absence of a control group, absence of knowledge of current medication and concomitant therapies, and absence of comprehensive testing prior to and throughout the program. No long-term follow-up data were present in charts; however, SOF were assumed to continue their career and deployment in adverse environments. No validated TBI scale or automated neuropsychiatric batteries were administered to program participants throughout the program. As post-concussive TBI symptoms overlap significantly with symptoms of PTSD, lack of TBI scale limits the specificity of the conclusions as to the efficacy of α-rTMS in this population. Additionally, formal neurocognitive evaluation as well as imaging of DMN connectivity via MRI were not captured, and should be gathered in future studies. Program safety is consistent with other applications of TMS, with noted initial elevations in reported headache and fatigue, however a randomized sham-controlled trial of α-rTMS is currently underway to corroborate these findings and further establish safety and efficacy of the treatment.

To our knowledge, this is the first chart review of an optimization program using α-rTMS deployed in a population of active-duty special forces personnel. Given the growing population of veterans with TBI history who show symptoms of PTSD and depression, prospective pre-retirement neuromodulation programs may aid in attenuating rates of mental health disorders and suicide post-deployment and in retirement. This data provides a demonstration of significant reduction in PTSD and depression symptoms and safety with the application of α-rTMS in active-duty special operations military personnel. Expansion of targeted neuromodulation programs could be impactful for military and civilian populations.

## Data availability statement

The data analyzed in this study is subject to the following licenses/restrictions: Dataset included deidentified patient chart data, this can be made available upon request to the corresponding author. Requests to access these datasets should be directed to MaryBeth Bailar-Heath drbailar@cespines.com.

## Ethics statement

Ethical approval was not required for the study involving humans in accordance with the local legislation and institutional requirements. Written informed consent to participate in this chart review was not required from the participants or the participants’ legal guardians/next of kin in accordance with the national legislation and the institutional requirements.

## Author contributions

MB-H: Writing – review & editing. RB: Writing – review & editing. DT: Writing – review & editing. CM: Writing – review & editing.

## References

[1] Raichle ME MacLeod AM Snyder AZ Powers WJ Gusnard DA Shulman GL. A default mode of brain function. Proc Natl Acad Sci. (2001) 98:676–82. doi: 10.1073/pnas.98.2.676 PMC14647 11209064

[2] Rona RJ Jones M Iversen A Hull L Greenberg N Fear NT. The impact of posttraumatic stress disorder on impairment in the UK military at the time of the Iraq war. J Psychiatr Res. (2009) 43:649–55. doi: 10.1016/j.jpsychires.2008.09.006 18950801

[3] Vogt D Smith B Elwy R Martin J Schultz M Drainoni ML. Predeployment, deployment, and postdeployment risk factors for posttraumatic stress symptomatology in female and male OEF/OIF veterans. J Abnorm Psychol. (2011) 120:819–31. doi: 10.1037/a0024457 21707125

[4] Wells A Welford M King P Papageorgiou C Wisely J Mendel E. A pilot randomized trial of metacognitive therapy vs applied relaxation in the treatment of adults with generalized anxiety disorder. Behav Res Ther. (2010) 48:429–34. doi: 10.1016/j.brat.2009.11.013 20060517

[5] Clancy CP Graybeal A Tompson WP Badgett KS Feldman ME Calhoun PS. Lifetime trauma exposure in veterans with military-related posttraumatic stress disorder. J Clin Psychiatry. (2006) 67:1346–53. doi: 10.4088/JCP.v67n0904 17017820

[6] Harpaz-Rotem I Hoff R. FY2021 Overview of PTSD Patient Population Data Sheet. West Haven, CT: Northeast Program Evaluation Center. (2022). VA Office of Mental Health and Suicide Prevention (11MHSP).

[7] Cooper AD Warner SG Rivera AC Rull RP Adler AB Faix DJ. Mental health, physical health, and health-related behaviors of U.S. Army Special Forces. PloS One. (2020) 15:e0233560. doi: 10.1371/journal.pone.0233560 32492027 PMC7269253

[8] Garcia A Kretzmer TS Dams-O'Connor K Miles SR Bajor L Tang X. Health conditions among special operations forces versus conventional military service members: A VA TBI model systems study. J Head Trauma Rehabil. (2022) 37:E292–8. doi: 10.1097/HTR.0000000000000737 34698680

[9] Garcia A Miles SR Reljic T Silva MA Dams-O’Connor K Belanger HG. Neurobehavioral symptoms in U.S. Special operations forces in rehabilitation after traumatic brain injury: A TBI model systems study. Military Med. (2021) 187:1412–21. doi: 10.1093/milmed/usab347 34591087

[10] Loignon A Ouellet M-C Belleville G. A systematic review and meta-analysis on PTSD following TBI among military/veteran and civilian populations. J Head Trauma Rehabil. (2020) 35:E21–35. doi: 10.1097/HTR.0000000000000514 31479073

[11] Moradi Y Dowran B Sepandi M. The global prevalence of depression, suicide ideation, and attempts in the military forces: a systematic review and Meta-analysis of cross sectional studies. BMC Psychiatry. (2021) 21:510. doi: 10.1186/s12888-021-03526-2 34654386 PMC8520236

[12] Scott JC Matt GE Wrocklage KM Crnich C Jordan J Southwick SM. A quantitative meta-analysis of neurocognitive functioning in posttraumatic stress disorder. Psychol Bull. (2015) 141:105–40. doi: 10.1037/a0038039 PMC4293317 25365762

[13] Kriesche D Woll CFJ Tschentscher N Engel RR Karch S. Neurocognitive deficits in depression: a systematic review of cognitive impairment in the acute and remitted state. Eur Arch Psychiatry Clin Neurosci. (2022) 273:1105–28. doi: 10.1007/s00406-022-01479-5 PMC10359405 36048295

[14] Akiki TJ Averill CL Wrocklage KM Scott JC Averill LA Schweinsburg B. Default mode network abnormalities in posttraumatic stress disorder: A novel network-restricted topology approach. Neuroimage. (2018) 176:489–98. doi: 10.1016/j.neuroimage.2018.05.005 PMC5976548 29730491

[15] Akiki TJ Averill CL Abdallah CG. A network-based neurobiological model of PTSD: evidence from structural and functional neuroimaging studies. Curr Psychiatry Rep. (2017) 19:81. doi: 10.1007/s11920-017-0840-4 28924828 PMC5960989

[16] Santhanam P Wilson SH Oakes TR Weaver LK. Effects of mild traumatic brain injury and post-traumatic stress disorder on resting-state default mode network connectivity. Brain Res. (2019) 1711:77–82. doi: 10.1016/j.brainres.2019.01.015 30641036

[17] Klimesch W Sauseng P Hanslmayr S. EEG alpha oscillations: the inhibition-timing hypothesis. Brain Res Rev. (2007) 53:63–88. doi: 10.1016/j.brainresrev.2006.06.003 16887192

[18] Bowman AD Griffis JC Visscher KM Dobbins AC Gawne TJ DiFrancesco MW. Relationship between alpha rhythm and the default mode network: an EEG-fMRI study. J Clin Neurophysiol. (2017) 34:527–33. doi: 10.1097/WNP.0000000000000411 PMC8428580 28914659

[19] Jann K Dierks T Boesch C Kottlow M Strik W Koenig T. BOLD correlates of EEG alpha phase-locking and the fMRI default mode network. NeuroImage. (2009) 45:903–16. doi: 10.1016/j.neuroimage.2009.01.001 19280706

[20] Knyazev GG Slobodskoj-Plusnin JY Bocharov AV Pylkova LV. The default mode network and EEG α oscillations: an independent component analysis. Brain Res. (2011) 1402:67–79. doi: 10.1016/j.brainres.2011.05.052 21683942

[21] Richard Clark C Veltmeyer MD Hamilton RJ Simms E Paul R Hermens D. Spontaneous alpha peak frequency predicts working memory performance across the age span. Int J Psychophysiol. (2004) 53:1–9. doi: 10.1016/j.ijpsycho.2003.12.011 15172130

[22] Samaha J Postle BR. The speed of alpha-band oscillations predicts the temporal resolution of visual perception. Curr Biol. (2015) 25:2985–90. doi: 10.1016/j.cub.2015.10.007 PMC4654641 26526370

[23] Sauseng P Klimesch W Doppelmayr M Pecherstorfer T Freunberger R Hanslmayr S. EEG alpha synchronization and functional coupling during top-down processing in a working memory task. Hum Brain Mapp. (2005) 26:148–55. doi: 10.1002/hbm.20150 PMC6871735 15929084

[24] Eidelman-Rothman M Levy J Feldman R. Alpha oscillations and their impairment in affective and post-traumatic stress disorders. Neurosci Biobehav Rev. (2016) 68:794–815. doi: 10.1016/j.neubiorev.2016.07.005 27435239

[25] Clancy KJ Andrzejewski JA Simon J Ding M Schmidt NB Li W. Posttraumatic stress disorder is associated with α Dysrhythmia across the visual cortex and the default mode network. eNeuro. (2020) 7 1–12. doi: 10.1523/eneuro.0053-20.2020 PMC7405069 32690671

[26] Schiena G Franco G Boscutti A Delvecchio G Maggioni E Brambilla P. Connectivity changes in major depressive disorder after rTMS: a review of functional and structural connectivity data. Epidemiol Psychiatr Sci. (2021) 30:e59. doi: 10.1017/S2045796021000482

[27] Clancy KJ Andrzejewski JA You Y Rosenberg JT Ding M Li W. Transcranial stimulation of alpha oscillations up-regulates the default mode network. Proc Natl Acad Sci U.S.A. (2022) 119:1–8. doi: 10.1073/pnas.2110868119 PMC8740757 34969856

[28] Taghva A Silvetz R Ring A Kim K.-YA Murphy KT Liu CY. Magnetic resonance therapy improves clinical phenotype and EEG alpha power in posttraumatic stress disorder. Trauma month. (2015) 20:e27360. doi: 10.5812/traumamon PMC4727473 26839865

[29] Roelofs CL Krepel N Corlier J Carpenter LL Fitzgerald PB Daskalakis ZJ. Individual alpha frequency proximity associated with repetitive transcranial magnetic stimulation outcome: An independent replication study from the ICON-DB consortium. Clin Neurophysiol. (2021) 132:643–9. doi: 10.1016/j.clinph.2020.10.017 33243617

[30] Lin Y-J Shukla L Dugué L Valero-Cabré A Carrasco M. Transcranial magnetic stimulation entrains alpha oscillatory activity in occipital cortex. Sci Rep. (2021) 11:18562. doi: 10.1038/s41598-021-96849-9 34535692 PMC8448857

[31] Corlier J Carpenter LL Wilson AC Tirrell E Gobin AP Kavanaugh B. The relationship between individual alpha peak frequency and clinical outcome with repetitive Transcranial Magnetic Stimulation (rTMS) treatment of Major Depressive Disorder (MDD). Brain Stimul. (2019) 12:1572–8. doi: 10.1016/j.brs.2019.07.018 31378603

[32] Petrosino NJ Cosmo C Berlow YA Zandvakili A van 't Wout-Frank M Philip NS. Transcranial magnetic stimulation for post-traumatic stress disorder. Ther Adv Psychopharmacol. (2021) 11:20451253211049921. doi: 10.1177/20451253211049921 34733479 PMC8558793

[33] Philip NS Doherty RA Faucher C Aiken E van ‘t Wout-Frank M. Transcranial magnetic stimulation for posttraumatic stress disorder and major depression: comparing commonly used clinical protocols. J Trauma Stress. (2022) 35:101–8. doi: 10.1002/jts.22686 PMC8581062 33973681

[34] Kim WS Paik NJ. Safety review for clinical application of repetitive transcranial magnetic stimulation. Brain Neurorehabil. (2021) 14:e6. doi: 10.12786/bn.2021.14.e6 36742107 PMC9879417

[35] Blevins CA Weathers FW Davis MT Witte TK Domino JL. The posttraumatic stress disorder checklist for DSM-5 (PCL-5): development and initial psychometric evaluation. J Trauma Stress. (2015) 28:489–98. doi: 10.1002/jts.22059 26606250

[36] Schalet BD Pilkonis PA Yu L Dodds N Johnston KL Yount S. Clinical validity of PROMIS Depression, Anxiety, and Anger across diverse clinical samples. J Clin Epidemiol. (2016) 73:119–27. doi: 10.1016/j.jclinepi.2015.08.036 PMC4928679 26931289

[37] Lam RW Lamy FX Danchenko N Yarlas A White MK Rive B. Psychometric validation of the Perceived Deficits Questionnaire-Depression (PDQ-D) instrument in US and UK respondents with major depressive disorder. Neuropsychiatr Dis Treat. (2018) 14:2861–77. doi: 10.2147/NDT PMC6211374 30464471

[38] Hedges LV. Distribution theory for glass’s estimator of effect size and related estimators. J Educ Stat. (1981) 6:107–28. doi: 10.3102/10769986006002107

[39] Lang AJ Wilkins K Roy-Byrne PP Golinelli D Chavira D Sherbourne C. Abbreviated PTSD Checklist (PCL) as a guide to clinical response. Gen Hosp Psychiatry. (2012) 34:332–8. doi: 10.1016/j.genhosppsych.2012.02.003 PMC3383936 22460001

[40] Kroenke K Stump TE Chen CX Kean J Bair MJ Damush TM. Minimally important differences and severity thresholds are estimated for the PROMIS depression scales from three randomized clinical trials. J Affect Disord. (2020) 266:100–8. doi: 10.1016/j.jad.2020.01.101 PMC7103541 32056864

[41] Zhou Y Milham MP Lui YW Miles L Reaume J Sodickson DK. Default-mode network disruption in mild traumatic brain injury. Radiology. (2012) 265:882–92. doi: 10.1148/radiol.12120748 PMC3504316 23175546

[42] Blumberger DM Vila-Rodriguez F Thorpe KE Feffer K Noda Y Giacobbe P. Effectiveness of theta burst versus high-frequency repetitive transcranial magnetic stimulation in patients with depression (THREE-D): a randomised non-inferiority trial. Lancet. (2018) 391:1683–92. doi: 10.1016/S0140-6736(18)30295-2 29726344

[43] Mysliwiec V McGraw L Pierce R Smith P Trapp B Roth BJ. Sleep disorders and associated medical comorbidities in active duty military personnel. Sleep. (2013) 36:167–74. doi: 10.5665/sleep.2364 PMC3543057 23372263

[44] Luxton DD Greenburg D Ryan J Niven A Wheeler G Mysliwiec V. Prevalence and impact of short sleep duration in redeployed OIF soldiers. Sleep. (2011) 34:1189–95. doi: 10.5665/SLEEP.1236 PMC3157660 21886356

[45] Committee on the Assessment of Ongoing Effects in the Treatment of Posttraumatic Stress Disorder Institute of Medicine. Treatment for posttraumatic stress disorder in military and veteran populations: Initial assessment. Washington (DC: National Academy Press (2012) 24830058

